# CD4^+^CD25^high^CD127^low/-^FoxP_3_^+^ Regulatory T-Cell Population in Acute Leukemias: A Review of the Literature

**DOI:** 10.1155/2019/2816498

**Published:** 2019-03-03

**Authors:** M. Niedźwiecki, O. Budziło, E. Adamkiewicz-Drożyńska, D. Pawlik-Gwozdecka, M. Zieliński, L. Maciejka-Kembłowska, T. Szczepański, P. Trzonkowski

**Affiliations:** ^1^Department of Pediatrics, Hematology and Oncology, Medical University of Gdansk, Poland; ^2^Clinical Immunology and Transplantology Unit at the Department of Immunology, Medical University of Gdansk, Poland; ^3^Department of Pediatric, Hematology and Oncology in Zabrze, Medical University of Silesia, Katowice, Poland

## Abstract

Regulatory T-cells (Tregs) are a very important subtype of lymphocytes when it comes to self-control in the human immunological system. Tregs are decisive not only in the protection against destruction of own tissues by autoimmune immunocompetent cells but also in the immunological answer to developing cancers. On the other hand, Tregs could be responsible for the progression of acute and chronic leukemias. In our study, we review publications available in the PUMED database concerning acute leukemia, with a particular emphasis on child's leukemias. The percentage of regulatory T-lymphocytes in peripheral blood and bone marrow was elevated compared to those in healthy individuals and correlated with progressive disease. Regulatory T-cells taken from children diagnosed with leukemia showed a higher suppressive capability, which was confirmed by detecting elevated levels of secreted IL-10 and TGF-beta. The possibility of pharmacological intervention in the self-control of the immunological system is now under extensive investigation in many human cancers. Presumably, Treg cells could be a vital part of targeted therapies. Routine Treg determination could be used to assess the severity of disease and prognosis in children with acute lymphoblastic leukemia. This proposition results from the fact that in some studies, higher percentage of Treg cells in peripheral blood was demonstrated. However, observations confirming these facts are scarce; thus, extrapolating them to the population of children with hematological malignancies needs to be verified in additional studies.

## 1. Introduction

Despite the rapid development of medicine, children are still diagnosed with cancers, which treatment with classical chemotherapy and radiotherapy does not give a chance for the permanent remission of the disease. In addition, current treatment implicates serious complications. Therefore, great hopes are bound with the development of such areas of medicine as cancer genetics and immunooncology.

Hence, it was decided to analyze the available literature on the research on the influence of Tregs on the initiation and progression of the most common childhood cancer—acute lymphoblastic leukemia (ALL) [1]. Generally, acute leukemias are heterogeneous hematological malignancies, diverse in terms of their clinical picture, phenotype, and detected genetic aberrations, as well as their final response to the applied treatment. Generation of carcinogenesis in hematological malignancies starts in the bone marrow, and infiltration of the lymphoblast can occur in every tissue and important organ of the human body [2]. Typical clinical manifestations of rapid leukemic hyperplasia are massive infiltration in organs and displacement of normal blood formation resulting in peripheral pancytopenia [3]. Due to the inhibition of normal hematopoiesis, the most common findings, when diagnosing childhood acute leukemia, are accompanying symptoms of anemia, leucopenia, and thrombocytopenia [4].

The most important part of the diagnostic process is immunophenotyping by flow cytometry, which reveals some main subtypes of leukemia: ALL B-cell or T-cell line and AML [5]. Except genetic aberration, biochemistry, and microscopic examination, distinguishing the source and differentiation stages of ALL by FC is crucial for the prognosis and clinical course of the disease [3, 6]. All of the above lead to the conclusion that we are ultimately interested in cancer cells rather than in the environment of cancers in the diagnostic and treatment process. Cells of the tumor's microenvironment are currently under intensive investigation [7, 8].

Residual nonmalignant T- and B-cells are in permanent cell-to-cell contact with blasts and are involved in active immune responses [9, 10]. Because of this, a very interesting clinical question occurs: what kind of physiological dependences is observed between blasts and regular progenitors of normal lines in the bone marrow? Is it the number of immune cells or its disturbed function or rather the general dysregulation of the immune system that is most important for the occurrence of cancer and final clinical effect of the treatment?

Regulatory lymphocytes stand clinically as a very interesting subpopulation of cells in a child's immune system. For instance, relatively small numbers of Tregs in the blood may condition the occurrence of autoimmune diseases, which due to the increasing incidence belong to “lifestyle diseases” [11]. Thus, for several years, a growing interest in their biological properties has occurred and clinicians have wondered whether they can also be used in the battle against cancer [11–13].

All authors agree on the fact that in immunodeficiency syndromes as well as during the period of immunosuppressive therapy, the risk of neoplastic disease occurrence is significantly higher [7, 12, 13]. On the other hand, it is also known that an efficient immune system definitely enhances the chance for permanent recovery from a neoplastic disease, by means of therapeutic protocols combining surgical procedures, chemotherapy, and radiotherapy. Hence, active antineoplastic immunotherapy is currently part of the standard procedure in the neoplasms like NHL or acute leukemias with a poor prognosis [14]. A rapid development of this branch of medicine is currently being observed. As a result of this, more and more new drugs consisting of monoclonal antibodies directed against neoplastic antigens occur (i.e., rituximab, alemtuzumab, or blinatumomab) [15–19].

Recent papers have demonstrated elevated levels of Tregs in lung, breast, pancreatic, ovarian, melanoma, digestive system cancers, CLL, T-cell ALL, and B-cell NHL [2, 20, 21]. This concerns both peripheral blood and neoplastic tissue, where a neoplastic proliferation is accompanied by higher than usual levels of regulatory lymphocytes. In some subtypes of neoplasms, the difference in the percentages may condition a response to chemotherapy and thus a prognosis of the disease [22, 23].

Yet, it is still unknown whether the increased percentage of Tregs is a cause or an effect of a neoplasm, particularly that an elevated percentage of Treg cells is observed even after achieving a remission and after completing treatment of acute nonlymphoblastic leukemia [24], as this would rather indicate that a neoplasm is more of an effect of immunological disorders caused by a mutated neoplastic cell.

The prognostic value and variation in the number of immune cell subpopulations differ from one histopathological subtype of neoplasm to another [25]. Whilst many papers report a prognostic significance of regulatory lymphocytes when solid tumors are considered, we still have poor knowledge about their meaning in hematological neoplasms, particularly in the neoplasms of a lymphatic system, which as a matter of fact stands as an integrated whole with lymphocytes themselves [26]. Previous research shows that the number of Treg lymphocytes may be either elevated or reduced [27, 28]. Similarly, a prognosis may be either favorable or adverse. It is known for example that the percentage of Treg cells is higher among patients suffering from CLL than that among healthy volunteers [29, 30]. This level correlates with advancement of the disease, the percentage of B-cells in the peripheral blood, and the level of LDH. In some papers, revelations that Treg cells may even control a neoplastic growth can be found [31]. They can support the fight against the developing cancer. This phenomenon has been observed in NHL, for example [32]. If this was confirmed, a hope for a better prognosis would appear.

The next interesting issue is a connection between Tregs and ALL among patients in the developmental age. This group of leukemias are characterized by a separate biology, clinical picture, and, first of all, a different prognosis [33]. As a potential target of immunotherapy, the problem above requires further, intense investigation [34]. This could contribute to the improvement of a prognosis with simultaneous reduction of toxic chemotherapy [29].

This review discusses some basic concepts in acute leukemia Treg interdependencies and the biological characteristics of regulatory cells. The scientific reports analyzed in the summary reveal clinically significant quantitative and qualitative abnormalities in regulatory cell subpopulations in acute leukemias.

However, the results obtained by the different authors are incomplete and cover only some of the important issues. Therefore, there is a need to undertake well-planned further research concerning larger groups of patients.

This paper also attempts to evaluate the possibility of improving the effectiveness of acute leukemia chemotherapy, by modifying Treg response to developing bone marrow proliferative disease.

## 2. Immunophenotype of Tregs

Biological significance of Tregs in the fight against cancer was noticed by researchers many years ago. It is reasonable to assume that the results of some of these studies are false. This is due to the fact that different authors use incorrect methods to determine the percentage of Tregs. It was only in recent years that the most appropriate phenotype of these cells has been identified and multicolor cytometry has been used to determine them. Nevertheless, the main problem remains because there are no reliable surface markers exclusively expressed on regulatory T-cells [31].

A recent study has demonstrated that CD4^+^CD25^high^CD127^low/-^FoxP_3_^+^ populations of T-cell are developmentally, phenotypically, and functionally different than CD4^+^ T-cells [35]. Therefore, a multicolor flow cytometry should be used to reliably evaluate and isolate the subpopulation of regulatory cells in peripheral blood and bone marrow [2]. Only this method allows to obtain reliable results of the cell population that we are interested in [35].

### 2.1. Surface Markers Expressed on Tregs

The decisive factor for the homeostasis and function of Treg population is IL-2 [10]. This cytokine is historically known as a T-cell growth factor. Furthermore, antigen CD25 is known as the alpha chain of the IL-2 receptor and is highly expressed on the surface of Tregs [36]. Besides CD25, Tregs also express cytotoxic T-lymphocyte-associated antigen-4 (CTLA-4), a glucocorticoid-induced TNF-alpha receptor (GITR) and member of the forkhead transcription factor (FoxP_3_) [25]. Like other antigens listed above, CD25 unfortunately is not exclusively expressed only on regulatory T-cells. It can be also found on the surface of activated effector T-cells [24].

Discovery of the intracytoplasmic FoxP_3_ was a milestone in the research of regulatory cells [37]. This transcription factor controls development, maintenance, and function of Tregs, which implicates the fact that a mutation of its genes leads to serious autoagressive or inflammatory diseases [12]. IPEX (immune dysregulation, polyendocrinopathy, enteropathy, X-linked) syndrome in humans may stand as an example here. Among others, we may list autoimmune manifestations in multiple endocrine organs, such a diabetes and thyroiditis, inflammatory bowel disease, and severe allergies [11, 13]. Another problem in determining the percentage of regulatory cells is the presence of the FoxP_3_ antigen also on the T-lymphoblasts [38].

Following this observation, an intensive molecular research has discovered gene-encoding key factors for the function of regulatory T-cells, including the FoxP_3_ gene. It has been proven that its most important part is the activating protein-1 (AP-1) [39, 40]. To sum up, subsequent studies have also revealed the enormous importance of the 6 nuclear factors of activated T-cell-binding sites (NF-AT) [40]. Then it was able to show that activation of AP-1 and other nuclear factors activates the receptor (TCR) found on naïve T-cells and the expression of FoxP_3_ which is crucial for the Treg function [9, 13, 41].

### 2.2. Production of the Cytokines by Tregs

Production of the cytokines by Tregs is determined by mentioned molecules. The proven inability of Tregs to produce and secrete proinflammatory cytokines (IFN-gamma, IL-4, and IL-2) is caused by the interaction of the FoxP_3_ molecule and nuclear factors—NF-*κ*B and NF-AT [42]. This is the main, but not the only, repression mechanism for the transcription of these cytokines by FoxP_3_-positive cells [31].

After the combination of NF-AT and FoxP_3_ molecules, there are two phenomena that are important for explaining the principles of regulatory cell functioning in the immune system [40]. First, upregulation of the CD25 antigen occurs, and second, the fusion of FoxP_3_ with the CTLA-4 molecule inhibits the CREB pathway. This happens through the production of cAMP after an interaction with the coactivating protein p300 [20].

Another Treg molecule worth mentioning is the surface antigen in the peripheral blood-DR MHC II (HLA-DR) [43, 44]. This particle appears in the activated regulatory lymphocyte and is associated with higher expression of Foxp_3_ and thus a stronger ability of immunosuppression of various immunocompetent cells [7, 43, 45]. Among them, it is worth to remember especially about CD4^+^ and CD8^+^ T-cells, dendritic cells, macrophages, B-cells, and natural killer cells (NK) [46].

Summarizing, several key proteins, cytokines, and metabolic pathways are responsible for the suppressor function of Tregs [20].

## 3. Subpopulation of Tregs and Their Origin (Figure 1)

In healthy individuals, according to some authors, Tregs represent around 6% of human CD4^+^ T-cells (2-10%). In other papers, the percentage of Tregs varies between 1 and 4% in peripheral blood [47]. A further study has demonstrated that this relatively small group of cells is not uniform [42]. There are two main subclasses among the T-regulatory cells. These two subclasses are distinguished depending on their origin and sites of generation. One of these are natural cells of thymic origin (natural Tregs (nTregs) and thymic Tregs (tTregs)), and the second group consists of regulatory T-cells induced in the peripheral lymphatic system (inducible Tregs (iTregs)) [9, 37, 48].

### 3.1. Natural Tregs (nTregs)

They are produced by the thymus expressing high levels of CD25, CTLA-4 receptors, and FoxP_3_. The latter is the most important factor for their development and function [25]. The alternative phenotype that characterizes nTregs is a combination of low to no expression of the IL-7 receptor (CD127) together with high expression of the IL-2 receptor (CD25) stated as CD127^low/-^CD25^high^ [49].

In order to isolate Tregs more precisely, molecules that could be linked only in this subset of cells have been searched for many years. For example, high expression of Helios and Neuropilin-1 has been recently described as the intracellular markers of nTregs which may also be helpful [50]. nTregs are produced from CD4^+^ thymocytes by a stimulation of the TCR receptor with autoantigens [51, 52]. This induces the expression of the CD25 antigen. In the next stage, IL-2 links to CD25 and transmits the signal via STAT5 inducing strong expression of FoxP_3_. As a result, the cells acquire a regulatory phenotype [51]. Immunosuppressive mechanisms in this subpopulation of Tregs are mainly cell-to-cell contact dependent, but the cytotoxicity and cytokines are also involved [41]. nTregs are mainly CD4^+^ T-cells but the expression of FoxP_3_ together with suppressive activity was also reported for CD8^+^ T-cells. Nevertheless, the latter population is much less known and described [47].

### 3.2. Induced or Adaptive Tregs (iTregs)

They are generated after activation from naive T-cells by stimulation of TCR at the periphery [12]. An antigen and high dose of TGF-beta are prerequisites in the process. Early on, these cells loose FoxP_3_ expression, produce IL-2 and IFN-gamma, and very quickly get old and undergo apoptosis. This subgroup consists of the following subsets:

1. Regulatory T-cells (Tr1) which secrete IL-10 [37]
2. T-helper (Th3) cells which secrete TGF-beta [52]
3. TGF-betta/IL-10 double-positive Treg [15]

### 3.3. Non-Treg Subclass of Immunosuppressive Lymphocytes

Apart from nTregs and iTregs, there are other subsets with suppressive capabilities in the immune system [31]. Suppressor function was found in the following subsets:

1. CD4^−^ and CD8^−^ T-cells with expression of the gamma/delta TCR
2. CD8^+^CD28^−^ cells with a suppressive effect

## 4. Mechanisms of Suppression

The most important part of the T-cell regulatory function is to maintain self-tolerance in the body [12]. What is known about Tregs (CD4^+^CD25^high^CD127^low/-^FoxP_3_^+^) is that they actively suppress both the pathological and physiological immune responses, mostly to self-tissues, by direct or indirect mechanisms [42]. It is precisely controlled, which allows the immune system to fight fungal, bacterial, and viral infections as well as cancer cells whilst at the same time, the host tissues are not affected. Nevertheless, there are many other mechanisms involved in the self-tolerance reaction [37, 49].

Evidence has shown that nTregs inhibit the activation and expansion of immunocompetent helper and cytotoxic T-cells [46]. However, other subsets can also be regulated. Activated nTregs suppress B-cells and their proliferation, immunoglobulin production, and class switch [37]. Innate immunity is also inhibited including NK, NKT cells, macrophages, and the function and maturation of DCs [49, 53]. The suppression of cytotoxic subsets by nTregs is of special importance in oncology as oversuppressed T-cells and NK cells allow for the faster spread of tumor cells [54–56].

### 4.1. Control of Treg-Mediated Suppression

Natural Tregs exert their activities in the immune system via TCR signals, costimulation receptor signals, and cytokine activity [37, 49]. One example of this may be the clinical use of high doses of IL-2, which, in combination with the antigen stimulation, causes a rapid increase of antigen-specific Tregs [35].

### 4.2. Cell-to-Cell Contact-Dependent Suppression

This is the first line of defense against autoimmunity [57]. The most important molecules taking part in this process belong to T-cell molecules. This immunological reaction is activated by regulatory T-cell molecules, such as CTLA-4 (CD152), LAG3, surface TGF-beta, and B7 family molecules (CD80 and CD86) [37]. Recent studies have shown the importance of the expression of FoxP_3_ with the coexistence of the CTL-4 molecule, for the function and significance of naive regulatory T-cells [41].

### 4.3. The Role of Cytokines

Several “*in vivo*” experiments support the role of IL-10, IL35, and TGF-beta in Treg-mediated suppression after self-antigen activation [36, 49]. For example, the action of IL-10 modulates the expression of immunosuppressive costimulatory molecules B7-H4 on the surface of DCs [12]. However, the production of IL-10 by Tregs depends on the microenvironment and differs between the organs of the human body [36]. Furthermore, Tregs present in the intestines produce a large amount of IL-10, most likely at the point of contact with a large number of foreign food antigens and the microbiome [58]. On the other hand, the lack of secretion of this cytokine by Tregs in the spleen is due to the lack of adequate stimulation [13, 31].

Another biologically relevant cytokine involved in the suppressive mechanism is TGF-beta [42]. The NOTCH1-HES1 axis in activated T-cells can be induced by Tregs with TGF-beta. Subsequent studies confirmed the key role of TGF-beta in the transformation of naive T-cells into regulatory cells, especially in the maintenance of natural Tregs [42]. To summarize, some experiments proved that TGF-beta, IL-2, and antigen stimulation can induce FoxP_3_ expression in naïve CD4^+^ T-cells [14]. Additionally, some authors have revealed the role of TGF-beta in cell-to-cell contact-dependent immunosuppression of NK-cells [36]. Interestingly, although IL-2 is not produced by Tregs, it is the most important cytokine necessary to maintain normal function and proliferation of Tregs [36]. In humans, IL-2 upregulates the expression of FoxP_3_. This phenomenon occurs with the help of STAT3- and STAT5-dependent pathways [35, 36, 59]. There are also cytokines which function to suppress the immunosuppressive potential of Treg cells. For example, IL-6 can induce T-cell resistance of regulatory cells [9, 41].

### 4.4. Cytotoxicity

Apoptosis of immunocompetent cells caused by Tregs is associated with two biologically active substances secreted by these cells—perforin and granzyme A. This process applies to both T- and B-lymphocytes, as well as monocytes and DCs [35, 60].

### 4.5. Glucocorticoid-Induced Tumor Necrosis Factor Receptor (GITR)

The next antigen which presented with higher expression on the surface of regulatory cells than on the other subclasses of T-cell lines is GITR. In the case of stimulation through this receptor, the appearance of T-cell resistance to the suppressive properties of Tregs is also observed [12].

### 4.6. Toll-Like Receptor and Inflammation

One of the most important antigens associated with the Treg immune response to pathogens is presented on their surface. These are TLRs which recognize PAMP [46]. Therefore, one of the most interesting phenomena observed in the acute phase of inflammation is the activation of Tregs through TLR2 [61]. This process induces the proliferation of Tregs with transiently impaired suppression. Also, TLR8 constitutively expressed on Tregs mediates enhanced antitumor immunity [61].

To summarize, the observations above present hope of possible intervention using immune regulation pathways in the near future. Controlling the number of Tregs, especially antigen-reactive Tregs and their suppressive activity, is a very promising option. Using this, we could adaptively control the upregulation or downregulation of physiological or pathological immune responses to tumor or pathogen antigens.

### 4.7. Therapeutic Perspectives in Humans [15, 62, 63]

#### 4.7.1. Reduction of Treg Number or Function

The possibility of lowering or increasing the number of regulatory lymphocytes, as well as their suppressive abilities, provides a great opportunity to improve the final therapeutic effect on the treatment of many diseases [10]. The greatest hope is to use such immunological therapies in the treatment of cancers [64]. This is due to the observation that natural Tregs in particular impair the effective antitumor response of the human immune system. Clinical and laboratory observations revealed that in many malignancies, Tregs vigorously infiltrate tumors together with elevated levels of these cells in peripheral blood and cancer tissues [51]. Subsequent studies have shown that therapeutic intervention in quantity and function of natural Tregs results in improved efficacy of chemotherapy and immunotherapy especially in hematological malignancies [10, 46]. The desired therapeutic effect can be obtained by blocking the GITR receptor with the help of specific monoclonal antibodies (anti-GITR mAbs) used systemically or locally [25].

#### 4.7.2. Increase of Treg Number or Function

In contrast to the higher peripheral blood levels of Tregs observed in cancerous diseases, there are lower levels of this subtype of cells in allergic reactions, inflammatory processes, and autoaggressive diseases [65, 66]. Another serious challenge is to achieve immune tolerance for transplanted organs [67, 68].

One of the most interesting treatments for these diseases might be the expansion of antigen-specific Tregs [34]. Unfortunately, the potential mechanism of action of Tregs in these diseases is still not fully understood [49]. It is assumed that this action depends on many biochemical factors, cellular relationships, physiological mechanisms, and pathological processes taking place in the microenvironment.

#### 4.7.3. Immunomodulation

Currently, in clinical practice, practically, every immunomodulating drug has an influence on the suppressor activity and number of Treg cells [15, 31]. Therefore, perhaps through the immunomodulating effect of these substances, the ultimate therapeutic effect on patients treated for acute leukemia might be influenced. The currently used immunomodulators include the following: anti-CD52 antibody, anti-thymocyte globulin, IL-1 inhibitor, anti-TNFa antibodies, anti-CD25 (IL-2R) antibody, anti-CD3 antibody, interleukin-2 (IL-2), mycophenolate mofetil (MMF), inhibitors, steroids, CTLA-4-Ig, tacrolimus, cyclosporin A, and calcineurin inhibitors (CNI) [65, 69–71].

## 5. Review of the Literature

Currently, there are relatively few reports on regulatory cells in acute leukemias. They mostly concern AML in adult patients with few scientific reports discussing this issue in children. It is also difficult to find studies assessing the function and predictive meaning of Tregs in hematological malignancies, as most papers assess the number of regulatory cells in the peripheral blood and cancer tissue of patients with various solid tumors. The publications analyzed in our work are presented in Table 1.

### 5.1. Childhood Acute Lymphoblastic Leukemia (ALL)

Some of the few researchers interested in the analysis of Tregs in acute childhood leukemia are Lustfeld et al. [1], who in 2014 analyzed populations of regulatory and nonregulatory cells in the bone marrow of children with acute B-cell and T-cell leukemias. The researcher stated that previously detected relationships between lymphocyte subpopulations and the age of a healthy volunteer can also be detected in the bone marrow of acute leukemia patients [47].

The most important observation emerging from this work was the detection of a correlation between the higher CD4/CD8 ratio at diagnosis and a favorable bone marrow (BM) response to chemotherapy on day 15 caused by non-Treg CD4^+^ cells. In addition, it was found that in patients with T-ALL there was a higher Treg BM proportion than in those with BCP-ALL. Summing up the study, Lustfeld et al. stated that CD4^+^ non-Treg cells in leukemic bone marrow at diagnosis may condition early remission of the disease but they did not find any influence of Tregs on the final prognosis in childhood ALL [1].

Salem et al. [28] in studies published in 2016 and 2018 assessed the percentage of Tregs in acute leukemia. The aim of the study was to analyze the number of Tregs and myeloid-derived suppressor cells (MDSCs) in pediatric populations of B-ALL patients (*n* = 45) in Egypt. The phenotypic cell populations analyzed during the study were HLA-DR-CD33^+^CD11b^+^ and CD4^+^CD25^+^CD127^−^. The result of this work was the observation of statistically significant increases in the numbers of MDSCs and Tregs in PB-ALL patients compared to healthy volunteers. The numbers of MDSCs and Tregs were statistically higher before induction of chemotherapy when compared to healthy controls. In contrast, authors noticed a significant reduction in the percentage of these cells in PB after initial chemotherapy.

Wu et al. explored the ratio of CD4^+^CD25^+^ and NK in the PB of patients under 18 years of age who suffered because of ALL before and after remission of the disease. This author postulates that the results have a higher number of CD4^+^CD25^+^CD127^−^ cells in patients at diagnosis and after remission compared to the control group. However, the number of Tregs decreased with the occurrence of remission [72].

### 5.2. Adult Acute Lymphoblastic Leukemia (ALL)

There are definitely more scientific reports on the importance of prognostic Tregs in adult ALL patients.

In 2015, Idris et al. [20] detected in his research significantly increased levels of Tregs in the MC of PB/BM patients with B-ALL. An additional discovery in this research was the positive correlation between patient's age and percentages of Tregs. In addition to assessing the percentage of regulatory cells circulating in the PB or BM in ALL in adults, the authors also tried to mark the level of activity of Tregs and compare it with those of healthy volunteers.

Wu et al. [2] explored the phenotype, the percentage of Tregs, and the expression of cytokines in adult patients with B-ALL and T-ALL. This mainly concerned IL-2, IL-10, and TGF-beta. Unfortunately, the size of the individual groups was too small and did not allow for extrapolation to the entire population. Nevertheless, the results obtained were very promising. They deduced that the main mechanism leading to the suppression in ALL is directly related to these three cytokines. Thus, lowering IL-2 concentration and increasing IL-10 and TGF-beta might be responsible for this mechanism of suppression. The authors showed in their study that IL-10 and TGF-*β* concentrations, in lymphoblast culture supernatants from ALL patients, were higher and, respectively, IL-2 concentrations lower compared to those in the controls. Furthermore, the percentage of Tregs was elevated in PB of ALL patients (*p* < 0.05). Elevated numbers of Tregs correlated with the altered levels of secreted cytokines and are indicative for a suppressive mechanism in the pathogenesis of ALL. It was a very interesting observation resulting directly from this research.

Bhattacharya et al. [27] investigated the role and regulation of Treg cells in B-ALL. In their study, ALL patients presented a lower number of CD4^+^CD25^+^ cells coexpressing a higher level of FoxP_3_, IL-10, TGF-*β*, and CD152/CTLA-4 than the healthy volunteers. These cells showed increased suppressive properties on CD4^+^CD25^−^ responder T-cells than normal. A very interesting finale conclusion resulted from this work. The suppressive capacity of the elevated number of regulatory cells in ALL increases with the severity of disease.

Another attempt to evaluate the importance of regulatory T-lymphocytes was that of Darwish et al. [73], who detected an increase in the total number of CD4^+^CD25^+^ T-cells compared to the total number of lymphocytes in the moment of diagnosis. The Absolute CD4^+^CD25^high^ number after induction of remission was much lower compared to those at the preinduction moment and the control. Additional observations showed a statistically significant increase in the percentage of CD4^+^CD25^high^ T-cells during a fever compared to preinduction. Therefore, the author stated that the frequency of Tregs in PB can be used as a biomarker to predict susceptibility to chemotherapy and prognosis of acute leukemia and take part in the immunological reaction to infection.

Similar results were obtained by Jarnicki et al. [8], who found normalization of the percentage of Tregs after hematological remission by patients with B-ALL diagnosis. Their suppressive activity finally was the same as those of healthy volunteers [8].

At present, the modern treatment of chemotherapy-resistant lymphoblastic leukemia (B-ALL and T-ALL) is based on the precise determination of the group with an unfavorable prognosis and personalization of chemical, immunological, and targeted molecular therapy [65]. An extremely important part of this treatment is therapy with the application of CD19-directed bispecific T-cell engager construct blinatumomab. These monoclonal antibodies combined with chemotherapy cause a hematological remission in 46.6% of patients with resistant B-ALL, resulting in a survival benefit when compared to intensive chemotherapy without immunotherapy [4].

Duell et al. [14], judged the role of Tregs in predicting the outcome of immunotherapy with blinatumomab in the population of patients with resistant leukemias. His data showed that blinatumomab responders had lower levels of Tregs in PB than nonresponders. Apart from Tregs, only bone marrow blast levels and LDH showed a weak prediction.

The mechanism of the blinatumomab action described above is extremely interesting. As a result of the infusion of bispecific mAbs, there is a chain of immunological phenomena causing activation of Tregs, which leads to the rapid release of IL-10 [74]. As a consequence, there is a decreased proliferation of CD8 lymphocytes. The consequence of this phenomenon is the reduction of antitumor effect of CD8^+^ T-cells [5]. Thanks to these studies, the meaning of Treg levels in PB for the efficacy of anticancer immunotherapy using blinatumomab is known. Evaluating the number of Tregs in PB patients with r/r ALL allows to estimate the group of patients with good response to the treatment. In addition, therapeutic Treg eradication might convert blinatumomab-resistant patients to responders [14].

Interesting conclusions were also drawn from other researches; Li et al. [75] found increased expression of FoxP_3_ on regulatory cells, in patients prior to treatment of acute lymphoblastic leukemia, in relation to healthy volunteers, and patients in a remission of the disease. This discovery suggested a suppressive role of this transcriptional factor in ALL.

### 5.3. Acute Myeloid Leukemia (AML)

In the current literature, there are reports of the importance of Tregs in adult AML. However, there are no scientific reports describing this problem among children.

In comparison, Wang et al. [60] described the possible contribution of Tregs in the impairment of the immune system in adult patients with AML. Additional subpopulations of T-cells were analyzed including CD4^+^CD25^−^ T-cells. The authors sorted the population from all mononuclear cells present in peripheral blood. At the next stage of the study, immunomodulatory and immunosuppressive properties of selected T-cell populations were assessed using proliferation and cytokine production assays.

As in the case of patients with acute lymphoblastic leukemia, adults with AML also had a higher percentage of CD4^+^CD25^high^ T-cells in PB. By evaluating the immunophenotype of these cells, it was determined that they were CD45^−^RA^−^, CD69^−^, CD45^−^RO^+^, CD95^+^, and intercellular CTLA-4^+^. The level of secreted cytokines IL-10 and TNF-alpha by CD4^+^CD25^high^ T-cells was low.

No secretion of IL-2, IL-4, IL-5, and IFN-gamma was noted at all. One of the extremely interesting functions of these cells is blocking the proliferation and production of IL-2 and IFN-gamma by CD4^+^CD25^−^ T-cells. However, after culturing both these cell populations with stimulation, it turns out that the function of the CD25^−^ cell population begins to resemble the function of CD25^+^ regulatory cells. Treg production of IL-10 is improved as result.

The comparison of Tregs in patients with AML and the control group showed that regulatory T-cells in AML were characterized by a higher proliferation index and a greater propensity for apoptosis. The most important conclusion from this work was the confirmation of a higher percentage of Tregs in PB patients suffering from AML, which is most probably related to the higher proliferative potential of these cells.

Interesting conclusions were obtained by Szczepanski et al. [24], who confirmed in their research a higher level of Tregs in PB and in “tumor tissue” in newly diagnosed untreated AML. The pretreatment numbers of Tregs predicted a response to chemotherapy. The study also showed that for the observed immunosuppression caused by Tregs, IL-10, TGF-beta1, and cell-to-cell contact are necessary. Proliferating autologous responder cells also influenced this phenomenon. There was a surprising result in the work saying that patients after chemotherapy in hematological and clinical remission everlastingly had higher percentage frequency of Tregs, which was inconsistent with the observations of other researchers. The conclusions from these studies were very interesting. Tregs in peripheral blood of AML adults cause suppression via contact-dependent and independent mechanisms, and Treg cells are resistant to conventional chemotherapy. Furthermore, a good response to chemotherapy was negatively correlated with the level of regulatory cells at the time of diagnosis. This observation was similar with those of other papers.

Interesting results were also obtained by research conducted by Shenghui et al. [44]. Observations included an increased percentage of CD4^+^CD25^high^CD127^low/-^ Tregs in PB patients with AML, as well as an even higher level in BM compared to PB.

Contrary to that by Szczepanski et al. [24] the research conducted by Shenghui et al. [44] showed that Treg levels were meaningfully higher in BM and PB at the moment of diagnosis and were reduced when the patients successfully finished chemotherapy. The levels of Tregs were higher again when the patients relapsed. Treg-mediated suppression was stronger in leukemias.

In the next stage of the study, the level of regulatory cells before therapy was analyzed in a group of patients who had resistant leukemia and died. CD4^+^CD25^high^CD127^low/-^ Tregs levels were higher in BM and PB than in patients with a good response to initial chemotherapy. According to some authors, similar results are obtained by examining PB and BM in healthy volunteers. Another cause of elevated lymphatic regulatory cells in the BM of children with acute leukemia may be their natural tendency to accumulate in this tissue [76].

This conclusion could be useful in clinical practice. Systematic determination of the level of regulatory cells during the treatment of patients suffering from acute leukemia may be of a great importance in predicting their final response to the treatment. Thanks to this method, the intensity of chemotherapy may be even more precisely adjusted to the aggressiveness of the diagnosed cancer disease.

### 5.4. Chronic Lymphocytic Leukemia (CLL)

Interestingly, D'Arena et al. [29] in 2011 have also shown in studies of CLL that the absolute number of Tregs is increased but the percentage is lower because of high WBC levels. The Treg number correlates with progressive disease (high WBC and LDH levels, B-cell lymphocytosis, and absolute CD38^+^ B-cell number). To summarize, The Treg level is higher in CLL patients and is similar to that of the group of patients with acute leukemia.

### 5.5. Phenotypes of Regulatory Cells Assessed by Individual Authors

Modern flow cytometers give the opportunity to assess accurately cell population, which may be identified based on many immunological parameters [46]. In the past, many scientific studies evaluating subpopulations of Tregs were based on the evaluation of the incomplete or even false phenotype of these cells. According to the analyzed literature, only a few research groups were able to fully evaluate the percentage of Treg cells in the peripheral blood of patients with acute leukemia based on the most appropriate phenotype—CD4^+^CD25^high^CD127^low/-^FoxP_3_^+^ T cells [27].

The need for multiparameter cytometry is caused by the presence of Treg-assessed antigens on other immune cells. As can be seen from the list of analyzed phenotypes of the regulatory lymphocyte population, many research groups did not include the most important antigen—FoxP_3_. In our opinion, this fact calls into question the reliability of the obtained results and makes it necessary to repeat some of the determinations based on the commonly recognized regulatory cell phenotype.

Analyzed lymphocyte populations in the reviewed works are as follows:

1. CD4^+^CD25^high^FoxP_3_^+^ T-cells [14]
2. CD4^+^CD25^high^CD127-FoxP_3_^+^ T-cells [27, 44]
3. CD4^+^CD25^high^ T-cells [2]
4. CD4^+^CD25^high^CD127^−^ T-cells [20, 77]
5. CD3^+^CD4^+^CD25^high^CD127^−^ T-cells [20]
6. HLA-DR-CD33^+^CD11b^+^ and CD4^+^CD25^high^CD127^−^ T-cells [1]
7. CD4^+^CD25^high^ T-cells, CD4^+^CD25^high^, and CD4^+^CD25^−^ T-cells [60]

## 6. Discussion

ALL is one of the most common childhood cancers with favorable treatment prognosis, but about 20% of cases suffer due to the relapsed and/or resistant disease [5]. Unfavorable prognosis is caused by serious complications of chemotherapy and risk factors. The most common genetic factors affecting the development of leukemias include DNA translocations, inversions, or deletions of genes involved in lymphocyte differentiation [78]. One example could be the *BCR-ABL* gene fusion that produces a specific protein kinase [79]. After the discovery of the protein kinase inhibitor named imatinib, the prognosis of ALL with fusion gene *BCR-ABL* improved [80]. Many authors believe that only targeted molecular therapy aided by immunological therapy can improve the effectiveness of the applied chemotherapy and increase the percentage of cured children with a reduction of intensity and toxicity of treatment [3].

Currently, pediatric and adult modern treatment methods of hematological malignancies contain in the therapeutic protocols few immunological procedures such us infusion of blinatumomab, rituximab, or alemtuzumab [81]. This type of immunological therapy radically changed prognosis in Burkitt's lymphoma and resistant/relapsed ALL [82]. Generally, monoclonal antibodies are focused on antigens present on the surface of cancer cells and on the activation of immunocompetent cells in cancer's microenvironment to kill them [4]. Therefore, the cancer cell microenvironment is one of the most meaningful elements in the initiation and further progression of hematological malignancies [21]. Especially, the quantity of tumor-infiltrating Tregs can significantly influence the prognosis in solid tumors and hematologic malignancies [83].

Tregs are one of the most interesting populations of immunologically competent cells engaged in fighting cancer [1, 83]. Other ones are NK cells, which have a great prognostic significance in myeloid malignancies [44, 53]. To date more and more evidence indicates that regulatory lymphocytes migrate to some particular sites in need of immune regulation [31, 76]. The percentage of Tregs found in blood and cancer tissue at diagnosis is indirectly indicative of the severity and malignancy of the cancer [77, 78].

An increased percentage of Tregs was noticed in breast, colon, and lung tumors. For example, Vigorè et al. [84] discovered the dependency of Treg percentage during the progression of cancer on the presence of metastases in different subtypes of solid tumors. Statistically, a higher percentage of regulator cells in PB was detected in patients with advanced cancer than those with no metastases. Similar dependencies should be expected in hematological cancers in children and adults. On the other hand, in the colorectal carcinoma, Tregs suppress bacteria-driven inflammation. Previous research has shown that this process promotes carcinogenesis [45]. In this situation, a higher percentage of Tregs correlates with better prognosis and is also an important risk factor.

Thus, it seems that similarly, tumor-infiltrating regulatory lymphocytes may have a great impact on prognosis also in acute leukemia [28]. Hence, they are a very interesting therapeutic option and they need to be determined for each cancer type separately, but especially in ALL and NHL [22]. In hematological malignancies, blasts and regulatory cells are in direct cell-to-cell contact all the time [35]. Both are part of the immunological system, and some similar properties of these immunocompetent cells may be expected. In acute leukemias, Hodgkin disease (HD), and non-Hodgkin lymphomas (NHL), regulatory T-cells were elevated in PB and correlated with the stage of the disease and the prognosis in most trials [26].

Intensive research carried out in recent years has generated new information about the development and biological characteristics of individual subtypes of leukocytes including regulatory T-cells [35]. This small population of T-cells is not uniform, but all their subtypes are very important when it comes to the immunological system. Treg depletion in mouse models of cancers improves endogenous immune-mediated tumor rejection [12]. That is why Tregs are very important in the battle against cancer but also play a relevant part of the autoaggressive reaction to their own tissue, which makes the whole problem quite complicated.

The importance of regulatory cells for immune tolerance has been confirmed also by the observations of Sánchez-Ramón et al. [85]. They noticed elevated percentages in the peripheral blood of pregnant women. During pregnancy, their percentage increases, which allows them to gain adequate tolerance to the developing cells and tissues of the growing fetus [85]. It is known that Tregs play a key role in the proliferation and activation of B-cells [86]. It is highly likely that Tregs have a similar function in the process of developing cancer. This is obviously an adverse effect, which finally leads to the inhibition of the desired response of the immune system.

Recent studies have shown a huge role of Tregs in promoting cancer progression and inhibiting the anticancer activity of the immune system [45]. The numbers and the suppressive activity of Tregs are increased in cancer patients as compared to healthy individuals. Some authors indicate a better prognosis associated with a higher percentage of regulatory lymphocytes, whilst others indicate quite the opposite [22, 24]. They show that prognosis also depends on the type of cancer and other unidentified biological factors [16]. There are many publications describing the prognostic significance of regulatory lymphocytes in various hematological cancers, such as CLL, CML, HD, NHL, and monoclonal gammapathies [7, 22, 24, 45].

A higher percentage of Tregs in human cancers predicts a worse immunological reaction not only to the viral infection but to the cancer's antigens also [20, 25]. Despite these observations, the significance of Tregs in the pathogenesis and the immunological reaction in acute leukemias is unclear [15]. As demonstrated by research done in recent years, cancer cells can stimulate Tregs to suppress the physiological response of the immune system to the developing disease. This pathological action reduces the efficiency of cancer immunotherapy [41].

Stasiak-Barmuta et al. [33] came to similar conclusions by putting forward a hypothesis on the promotion of leukemic hyperplasia through activated Tregs. An interesting discovery of this author was not only the abnormal expression of surface antigens but also the pathological secretion of cytokines [33].

However, there are few publications describing the importance of Tregs in ALL, in particular those occurring in developmental age. Bhattacharya et al. [27] point to an increased percentage of Tregs in patients with B-line ALL. In their study, a correlation with the disease stage was confirmed [27]. Lustfeld et al. suggested that an elevated percentage of CD4^+^ T-cells amid residual BM T-cells in ALL is associated with a favorable prognosis for early hematological remission and in turn affects the final prognosis [1].

All authors of the publications analyzed in our study detected an increased percentage of regulatory cells in PB and/or BM of patients with acute leukemia [87]. We have no doubt about this fact. However, our concerns are caused by the various panels of antibodies used in FC to establish in detail the percentage of Tregs [2, 20, 28]. In some studies, the panel did not contain the basic parameter for determining Tregs—FoxP_3_ [1, 2, 9, 14, 20, 77]. Generally, Treg cells are defined on the basis of combined expression of CD4, CD25 at high density, low expression of the CD127, and FoxP_3_ [27, 75]. Nuclear factor FoxP_3_ is the most important part in the specification of Treg cells, so both the percentage and their general number among CD4-positive cells in the blood and cancer tissue were established according to this factor. Research carried out by Li et al. indicated an immunosuppressive function of this transcriptional factor in ALL [75]. However, theses with an elevated percentage of regulatory cells in acute leukemias defend the fact of obtaining repetitive results despite the use of different antibody panels. These interesting results were obtained despite the small groups of patients, measuring the percentage of regulatory cells in PB and/or BM, as well as in heterogeneous age groups of patients [73]. Treg cells were analyzed in a population of PB mononuclear cells or BM cells only in selected studies which prevents the generalization of results across the entire population of patients with acute leukemia [20].

Very interesting are the observations of a lower percentage of Tregs after the applied cytostatic treatment, which may support their importance in the pathogenesis of acute leukemias [44]. The different results were obtained by Szczepanski et al., who detected an identical percentage before and after chemical treatment [24]. This observation could support the argument that Tregs are resistant to chemotherapy.

In acute leukemias, elevated percentages of Tregs in PB and/or BM are also associated with their increased suppressive properties [2, 24, 33, 44, 60]. Also, in this case, their suppressive properties were evaluated by different methods, which makes it necessary to repeat these tests on a larger group of patients using standardized diagnostic methods. Another problem hindering a reliable analysis of the data obtained on small groups of patients suffering from acute leukemia is the fact that age has a significant influence on the lymphocyte subpopulations present in PB and BM [1].

Another problem in the reliable analyses of the data from Treg research is the presence of a marker characteristic of them also on other cells [38]. The most surprising is the occurrence of FoxP_3_ antigen on T-ALL cells as well as on myeloblasts and macrophages [24, 38]. Because of that, the author concludes that Tregs can be an important factor in relapsed AML and they can cause problems in their determination by means of flow cytometry. As shown by some studies, the percentage of Tregs may be a good indicator of the response to chemotherapy and the immunotherapy used [14]. Blinatumomab responders had lower levels of Tregs in peripheral blood than nonresponders in resistant/relapsed ALL [14].

The importance of Tregs in the development and promotion of hematological tumors, confirmed by the researchers, increases the interest in new therapies including manipulations of these cells. Treg inhibition and/or depletion, the latter using monoclonal antibodies directed against antigens on the surface of the regulatory cells such as CD25, is currently under investigation [41].

Except Treg depletion, tumor immunotherapy consists of vaccination which causes CTLA-4 blockade [83]. Another example is Ontak, which is a recombinant protein containing IL-2 and diphtheria toxin. This particular protein links to the antigen CD25 present on the cells' surface of T-cell leukemia/lymphoma. On the other hand, a high expression of CD25 is also observed among Tregs [10, 88].

The prognostic value of Tregs was confirmed by Massa et al. [89] also for primary myelofibrosis, where the use of targeted treatment with a *JAK 1/2* inhibitor (ruxolitinib) leads to a profound and long-lasting reduction in the frequency of circulating Tregs. The *JAK 1/2* inhibitor inhibits the release of sIL-2R alpha, an inflammatory cytokine produced by Tregs. This confirmed previous observations about the interdependence of various mechanisms of cancer in the human body. Therefore, the time is coming to apply various antitumor immunotherapy methods, both passive and active, supported by targeted therapy of kinase inhibitors [90, 91]. This approach, like the introduction of multidrug chemotherapy for the treatment of pediatric ALL by the BFM group, should bring further progress and hope for children with poor-prognosis ALL.

## 7. Conclusion

Tregs are a group of cells with fundamental function in maintaining immunological homeostasis in health and disease including acute leukemias. Depending on the cancer subtype, they can support the fight against the developing cancer, or on the contrary, they can promote the progression of blastic cells. Manipulations involving Tregs are potentially an interesting therapeutic option and perhaps may be used to enhance the effect of antitumor chemotherapy.

Extrapolation of the obtained results of the research on the population of patients diagnosed with acute leukemia is impossible due to the small groups of assessed patients and the use of a different research methodology.

In addition, the researchers evaluated the selected parameter in the PB or BM in the mononuclear cell population or all white blood cells based on the patchy phenotype of the Tregs.

Extensive clinical and laboratory studies are now warranted to validate these findings and determine their practical use and clinical implications. Future clinical trials as well as laboratory findings may result in a new scope, where the manipulation of Treg population brings long-term remission of hematological malignancies.

## Figures and Tables

**Figure 1:**
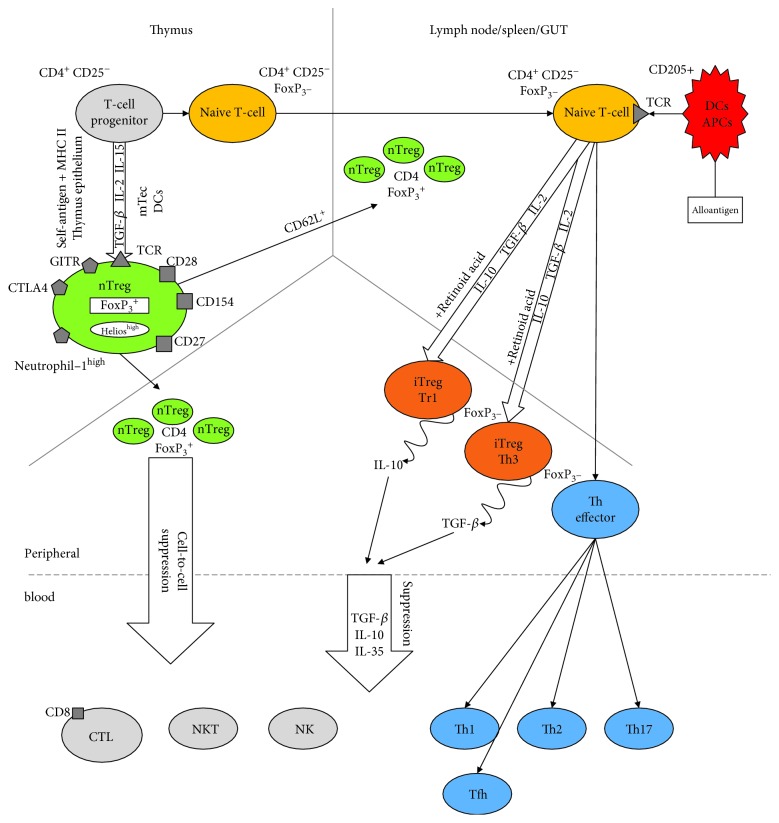
Ontogeny of Tregs in the body.

**Table 1:** Review of the literature—Tregs in acute leukemia in child and adult patients.

ALL/AML/patients	BM/PB sample	Frequency of Tregs	Function of Tregs	Impact on prognosis	Reference
B-ALL/controls (adults)	BM/PB	Increased	Increased suppressive capability	Correlation with disease progression Returned to the normal level in remission	Bhattacharya et al., 2014 [27]
B-ALL, T-ALL/controls (adults)	PBMC	Increased	Tregs suppress immune function in ALL through the downregulation of IL-2 and upregulation of IL-10 and TGF-beta	Not evaluated	Wu et al., 2012 [2]
B-ALL/controls (adults, child)	BMMC/PBMC	Increased	Not evaluated	Not tested Correlation with age	Idris et al., 2016 [20]
B-ALL, T-ALL (child)	BM	Increased, higher in T-cell than B-cell ALL	Not evaluated	CD4^+^ non-Treg cells may have a role in early response to treatment	Lustfeld et al., 2014 [1]
ALL/AML (adult)	PB	Increased	Not evaluated	Tregs may be used as a biomarker for predicting sensitivity to chemotherapy and prognosis	Darwish et al., 2015 [73]
B-ALL (child)	PB	Increased, lower after chemotherapy	Not evaluated	Not evaluated	Salem et al., 2016 [28]
ALL (child)	PB	Increased	Activation of Treg cells is one of the mechanisms of immunosuppression in ALL	Not evaluated	Stasiak-Barmuta et al., 2009 [33]
ALL (child)	PB	Increased	The increase of Tregs and decrease of NK cells indicate that the function of NK cells may be depressed	Treg T-cells play a role in occurrence and development of leukemia and are involved in downregulating NK cell function	Wu et al., 2010 [72]
ALL (adult)	PB	Increased	The serum derived from the ALL patients can convert CD4^+^CD25^−^ T-cells to CD4^+^CD25^+^ Tregs, which might be the important reasons for immunosuppression	Not evaluated	Li et al., 2007 [75]
AML (adults)	PBMC/BMMC	Increased	Inhibition of proliferation and cytokine production (IL-2, IFN-gamma) and improved IL-10 production	Predicted response to chemotherapy	Wang et al., 2005 [60]
AML/controls (adults)	PB/BM	Increased before and after induction	IL-10, TGF-beta1, and cell-to-cell contact are necessary for higher Treg-mediated suppression	Predicted better response to chemotherapy	Szczepanski et al., 2009 [24]
AML (adults)	PB/BM	Increased, particularly high in R/R AML	Tregs may play a role in pathogenesis of AML	Sequential measurement of Treg frequency may have a clinical value in the evaluation of therapeutic effects and clinical outcome Associated with poor prognosis	Shenghui et al., 2011 [44]
AML (adults)	PB	Increased	Treg cells may play a suppressive role in host antitumor immune response	Tregs may be a biomarker for predicting sensitivity to chemotherapy and prognosis	Yang et al., 2013 [77]

## Abbreviations

ALL: Acute lymphoblastic leukemia
AML: Acute myeloid leukemia
APCs: Antigen-presenting cells
BCP-ALL: B-Cell precursor acute lymphoblastic leukemia
cAMP: Cyclic adenosine monophosphate
CD4: Cluster of differentiation 4, protein found on T-helper cells, monocytes, macrophages, and dendritic cells
CD25: Cluster of differentiation 25, the alpha chain of the IL-2 receptor
CD27: Cluster of differentiation 27, a member of the tumor necrosis factor receptor superfamily
CD28: Cluster of differentiation 28
CD80: Cluster of differentiation 80, protein found on dendritic cells, activated B-cells, and monocyte
CD86: Cluster of differentiation 86, protein found on antigen-presenting cells
CD127: Cluster of differentiation 127; IL-7R alpha
CD154: Cluster of differentiation 154, a marker of activation expressed on T-cells
CLL: Chronic lymphocytic leukemia
CREB: cAMP response element-binding protein
CTL: Cytotoxic T-lymphocyte
CTLA-4: Antigen CD152, the cytotoxic T-lymphocyte-associated antigen 4
DCs: Dendritic cells
FC: Flow cytometry
FoxP_3_: Forkhead transcription factor
GITR: Glucocorticoid-induced tumor necrosis factor receptor family-related protein
GUT: Intestine
HD: Hodgkin's disease
Helios: A member of the Ikaros transcription factor family
HLA-DR: Human leukocyte antigen-DR isotype
IGF gamma: Interferon gamma
IL: Interleukin
JAK: Janus kinase
LAG3: Lymphocyte-activation gene 3
LDH: Lactate dehydrogenase
mAbs: Monoclonal antibodies
MC: Macrophages
MDSCs: Myeloid-derived suppressor cells
MHC II: Class of major histocompatibility complex molecules II
Mtec: Medullary thymic epithelial cell
Neuropilin-1: Type 1 transmembrane protein
NHL: Non-Hodgkin lymphoma
NK: Natural killer
NKT cells: Natural killer T-cells
NOTCH-1: A transmembrane molecule, the expression of which is required in the T-cell commitment of lymphoid progenitors
PAMPs: Pathogen-associated molecular pattern
PB: Peripheral blood
r/r ALL: Refractory/relapsed acute lymphoblastic leukemia
STAT 3: A signal transducer and activator of transcription 3
STAT 5: A signal transducer and activator of transcription 5
TCR: T-cell receptor
Tfh: Follicular B-helper T-cells
TGF-beta: Transforming growth factor *β*_1_
Th1: Type 1 T-helper cells
Th2: Type 2 T-helper cells
Th3: T-helper 3 cells
Th17: T-helper 17 cells
TLRs: Toll-like receptors
TNFa: Tumor necrosis factor alpha
Tr1: CD4^+^Foxp_3_^−^ type 1 regulatory T-cells
WBC: White blood cells.
